# Proteomics: Potential techniques for discovering the pathogenesis of connective tissue diseases-interstitial lung disease

**DOI:** 10.3389/fimmu.2023.1146904

**Published:** 2023-03-29

**Authors:** Yinlan Wu, Yanhong Li, Yubin Luo, Yu Zhou, Xiuping Liang, Lu Cheng, Tong Wu, Ji Wen, Chunyu Tan, Yi Liu

**Affiliations:** ^1^ Department of Rheumatology and Immunology, West China Hospital, Sichuan University, Chengdu, China; ^2^ Rare Diseases Center, West China Hospital, Sichuan University, Chengdu, China; ^3^ Institute of Immunology and Inflammation, Frontiers Science Center for Disease-related Molecular Network, West China Hospital, Chengdu, China; ^4^ Department of Respiratory and Critical Care Medicine, Chengdu First People’s Hospital, Chengdu, China

**Keywords:** proteomics, interstitial lung disease, connective tissue diseases-interstitial lung disease, differentially expressed proteins, rheumatoid arthritis, systemic sclerosis, idiopathic inflammatory myopathies

## Abstract

Interstitial lung disease (ILD) is one of the most serious lung complications of connective tissue disease (CTD). The application of proteomics in the past decade has revealed that various proteins are involved in the pathogenesis of each subtype of CTD-ILD through different pathways, providing novel ideas to study pathological mechanisms and clinical biomarkers. On this basis, a multidimensional diagnosis or prediction model is established. This paper reviews the results of proteomic detection of different subtypes of CTD-ILD and discusses the role of some differentially expressed proteins in the development of pulmonary fibrosis and their potential clinical applications.

## 1 Introduction

Interstitial lung disease (ILD) is a group of common heterogeneous lung diseases characterized by inflammation and fibrosis of lung tissue, which is usually progressive and fatal (1, 2). ILD is one of the most common complications of connective tissue disease (CTD), and studies have shown that approximately 32% of CTD patients have ILD (3). Approximately 30% of ILD cases are associated with CTD, and approximately 15% of ILD patients develop CTD after diagnosis (4). Therefore, the concept of CTD-ILD was proposed to include any diffuse parenchymal lung disease in patients with CTD (5). The CTDs associated with ILD include systemic sclerosis (SSc), rheumatoid arthritis (RA), dermatomyositis (DM), polymyositis (PM), Sjogren’s syndrome (SS), and systemic lupus erythematosus (SLE) (5, 6). ILD in CTD is associated with a number of adverse outcomes, such as reduced quality of life, disability, and death (7–9). Notably, the etiology and pathogenesis of different subtypes of CTD-ILD are not fully understood, hindering accurate diagnosis and precise treatment in clinical practice. Therefore, it is critical to explore the potential pathological mechanism and new diagnostic and therapeutic targets of each CTD-ILD subtype.

Proteomics is a relatively mature “omics” field that refers to the study of protein expression, quantification, localization, function, changes in molecular form, posttranslational modification, conformation, chemical modification, and protein−protein interactions at specific times and under specific conditions (10). In particular, the advent of gel-based and gel-free proteomics techniques and advances in mass spectrometry (MS) allow rapid, unbiased, systematic, and high-throughput identification and quantification of samples of a variety of complex protein mixtures, such as bronchoalveolar lavage fluid (BALF), serum, and lung tissue (11). Therefore, proteomics technology is suitable for exploring the mechanism of complex diseases such as CTD-ILD and helping to clarify pathogenesis and pathological changes, which is vital for establishing accurate clinical diagnosis and prognosis models and identifying therapeutic targets for different CTD-ILD subtypes. Therefore, we summarized the results of proteomic studies on different types of CTD-ILD (**Table 1**), in addition to the functions of several important proteins and their correlation with CTD-ILD (**Table 2**), to provide references for future basic research or clinical application of CTD-ILD.

**Table 1:** Proteomics study of various CTD-ILD.

Study ailment	Sample type	Control group	Changes in the proteome	Important protein	Reference
**RA-UIP**	BALF from patients	RA-OP	There was no significant difference in protein abundance, but the expression levels of the six proteins were significantly different between the UIP and OP	Immunoglobulin κ chain C region, gelsolin, α-1 antitrypsin, CRP, Haptoglobin β, SFTPA	(12)
**RA-ILD**	Serum from patients	RA Without ILD	There were 234 DEPs, of which 16 were upregulated and 5 were downregulated in both RA-ILD and IPF	PIANP, SLPI, CCL1810, IL-1711, CXCL12, CCL5, FGF, LGALS3, galectin-3, MMP7	(13)
**RA-ILD**	Serum from patients	RA Without ILD	Thirteen protein peaks were detected that were all downregulated in RA-ILD patients	The best tree was established to distinguish RA-ILD from RA by protein peak mass-to-charge value	(14)
**SSc-ILD**	BALF from patients	IPF and sarcoidosis	There were quantitative but not qualitative differences in protein composition among the three diseases, and the BALF protein composition of SSc-ILD was between that of sarcoidosis and IPF	α2-macroglobulin, Prothrombin, Cal B	(15)
**SSc-ILD**	BALF from patients	SSc without ILD	Three proteins increased and two decreased	α2-macroglobulin, α1-antitrypsin, SFTPA, HSP, GST	(16)
**SSc-ILD**	Fibroblasts in lung tissue from patients	Mild asthma	——	ED-A fibronectin, α-SMA	(17)
**SSc-ILD**	Fibroblasts in BALF from patients	Mild asthma	There were 24 DEPs, of which 13 DEPs showed more than 2-fold expression difference	RanBP1, ERp60, GSTP1-1	(17)
**SSc-ILD**	BALF from patients	IPF/Sarcoidosis/PLCH	There were 77 kinds of DEPs in each group. The levels of most kinds of DEPs in BALF of SSc-ILD patients were higher than those of IPF patients and lower than those of PLCH	Plastin 2, Annexin A3, 14-3-3ϵ, S10A6, GSTP1, PRDX1, ANXA3	(18)
**SSc-ILD**	BALF from patients	SSc without ILD	There were 11 DEPs or protein fragments	GSTP, SOD, Cystatin SN, α1-acid glycoprotein, haptoglobin-α chain, Cal B, Cytohesin-2, Calumenin, mtDNA TOP1	(19)
**SSc-ILD**	BALF from patients	Normal controls	Twenty-one kinds of DEPs were found, and the levels of most of them were increased in BALF of SSc-ILD	A1AT, APOAI, Angiotensinogen, GSTP1 and 14-3-3, S100A6, C3a, haptoglobin, CERU, B2MG, SFPA2, PRDX1, MANR1	(20)
**dcSSc**	Plasma from patients	lcSSc/Normal controls	S100A8/A9 was significantly increased in SSc and correlated with PF in lcSSc. In diffuse SSc, S100A8/A9 levels were similar with or without PF	S100A8/A9	(21)
**SSc**	Plasmacytoid dendritic cells from patients	Normal controls	Plasmacytoid dendritic cells in SSc showed distinctive peak patterns. SSc patients with higher CXCL4 levels have earlier evidence of PF, significantly faster decline in lung function, and a higher prevalence of PF	CXCL4, CTAP-III, S100A8/9, lysozyme	(22)
**SSc-ILD**	EVs precipitated from plasma from patients	Normal controls	EV proteins of SSc-ILD are mainly involved in platelet activation, cell adhesion, and immune responses	MT-A	(23)
**Patients who died of scleroderma**	Pulmonary fibroblasts from patients	Patients who died from nonpulmonary diseases	CTGF, 9 of which have not been reported in PF	Pro-α collagen, Caldesmon, Prolyl 4-hydroxylase β-subunit, IQGAP1, Cytoskeleton-associated protein-4, Ezrin, Moesin, Vinculin, BiP glucose-regulated protein, ER-60 protease, HNRPU, Valosin-containing protein, Stress-induced phosphoprotein-1	(24)
**SSc**	Extracellular matrix of fibroblasts *in vitro*	IPF/Normal controls	There was a high overlap between SSc and IPF matrix proteins, and the soluble matrix proteins of SSc and IPF were significantly different from those of healthy controls	PLOD2, LUM, POSTN, IGFBP5, GREM1, SPARC	(25)
**SSc**	Fibroblasts *in vitro*	Normal controls	A total of 155 proteins were directly ubiquitinated after KLHL42 knockdown, and 291 proteins were found only after KLHL42 knockdown	PPP2R5ϵ	(26)
**SSc-ILD**	Lung cell scaffold *in vitro*	Normal controls	Periostin in SSc was similar to the changes previously reported in decellularized IPF lung cells, but multiple proteins were more specific in SSc.	Periostin, Fibulin 3, TINAG-like 1, and Elastin	(27)
**ASS/IIM**	Serum from patients	Normal controls	IgG fragments can distinguish ASS patients with ILD from those without ILD	Fc-agalactosylated glycan	(28)
**PM/DM-ILD**	BALF from patients	AS-ILD/Overlap	There were 24 specific protein spots among the three groups, 9 spots were only present in PM/DM-ILD, 3 spots were only present in AS-ILD, and 12 spots were only present in overlap syndrome ILD	Gelsolin, Vimentin, Human myotonic dystrophy protein kinase, cofilin 1, Pyruvate kinase, al B, Peroxiredoxin 1, Coenzyme Q10, D-3-Hydroxybutyrate dehydrogenase and β-globin	(29)
**CTD-ILD**	BALF from patients	The healthy lung of CAP	Sixty-five DEPs were upregulated and 67 DEPs were downregulated	SFTPD, CADM1, ACSL4, SIL1, WIPF1, VCAM-1, JAML, GALNT1, NDPKB, CPB2	(30)
**Progressive ILD**	Plasma from patients	Nonprogressive ILD	Thirty-one proteins were associated with progressive fibrotic ILD, and a progressive pulmonary fibrosis risk assessment model consisting of 12 proteins was established by LASSO analysis	AGER, CST7, CXCL10, DPP10, FASLG, ITGB6, KRT19, MEPE, PLAUR, PNPT1, TNFSF11, WFIKN2	(31)

SFTPD, surfactant protein D; CADM1, cell adhesion molecule 1; ACSL4, long-chain fatty acid CoA ligase 4; WIPF1, Wiskott–Aldrich syndrome protein interacting protein family member 1; VCAM-1, vascular cell adhesion molecule 1; JAML, junctional adhesion molecule-like; GALNT1, polypeptide N-acetylgalactosaminyltransferase 1; NDPKB, nucleoside diphosphate kinase B; CPB2, Carboxypeptidase B2.

**Table 2:** Summary of important proteins.

Protein name	Disease type	Sample type	Effector	Related proteomic studies
Paired immunoglobulin-like type two receptor-associated neural protein	RA-ILD	Serum from patients	Regulation of neutrophils mediates inflammatory responses	(13)
Secretory leukocyte peptidase inhibitor	RA-ILD	Serum from patients	Resistance to neutrophil elastase destruction	(13)
C-gelsolin	RA-UIP	BALF from patients	Involved in caspase-3 related apoptosis process	(12)
N-gelsolin	RA-UIP	BALF from patients	Involved in caspase-3 related apoptosis process	(12)
Glutathione S-transferase P	SSc-ILD	BALF from patients	Repair membrane phospholipid damage and inhibit microsomal peroxidation	(16, 19)
14-3-3ϵ	SSc-ILD	BALF from patients	Guide the plasticity of macrophages	(18, 20)
Transthyretin	SSc-ILD	BALF from patients	Involved in the activation of endoplasmic reticulum stress	(15, 18, 20)
S100A8/Calprotectin	SSc-ILD	Plasma from patients	Induced the proliferation of fibroblasts	(21)
	SSc-ILD	BALF from patients		(19)
CXCL4	SSc-ILD	The supernatant of dendritic cells from patients	Associated with decreased lung function in patients	(21)
MT-A TP6	SSc-ILD	EV in plasma from patients	Involved in platelet activation, cell adhesion, and immune responses	(23)
Fc-glycans agalactosylated IgG	IIM-ILD	Serum from patients	Regulating the immune system	(28)
Gelsolin	PM/DM-ILD	BALF from patients	Improved airway mucus viscosity and preserves the intrinsic antimicrobial activity of airway surfaces	(29)
	RA-ILD			(12)
Calgranulin B	PM/DM-ILD	BALF from patients	Involved in the recruitment of leukocytes to sites of inflammation	(29)
	SSc-ILD			(19)
Surfactant protein D	SSc-ILD	Serum from patients	Involved in the innate immune system	(30)
	RA-ILD	Serum from patients		(32)
	PM/DM-ILD	Serum from patients		(32)
Cell adhesion molecule 1	CTD-ILD	Serum from patients	Regulation of human lung mast cell adhesion receptors to lung fibroblasts	(30)
SIL1	CTD-ILD	Serum from patients	Nucleotide exchange factors involved in ER stress	(30)
N-sulfoglucosamine sulfohydrolase	CTD-ILD	Serum from patients	Desulfation of glycosaminoglycan chains on proteoglycans	(30)

SSc, systemic sclerosis; ILD, interstitial lung disease; RA, rheumatoid arthritis; PM/DM, polymyositis/dermatomyositis; CTD, connective tissue disease; UIP, usual interstitial pneumonitis; BALF, bronchoalveolar lavage fluid; EV, extracellular vesicles; IIM, idiopathic inflammatory myopathy.

## 2 Proteomics studies in different CTD-ILD subtypes

### 2.1 Proteomics studies in RA-ILD

RA is a chronic systemic autoimmune and inflammatory disease characterized by synovitis and vasculitis (33). RA-ILD is a common and serious complication of RA. Approximately 40% to 58% of patients with RA develop ILD (34, 35), and the 5-year mortality rate is estimated at 35% (36). Therefore, it is crucial to determine the etiology, pathogenesis, and prognostic factors of RA-ILD, which can be assisted by proteomics.

To investigate the pathogenesis of RA-ILD, Wu et al. compared the serum proteome of RA patients with or without ILD using SOMA scan analysis and found 234 differentially expressed proteins (DEPs). These DEPs provide a good direction to study the pathogenesis of ILD in patients with RA (13). In clinical practice, RA-ILD is associated with various pulmonary imaging manifestations, such as usual interstitial pneumonia (UIP), nonspecific interstitial pneumonia (NSIP), organizing pneumonia (OP), and lymphocytic interstitial pneumonia (LIP), with each having its treatment and prognosis (37). Suhara et al. analysed the BALF of patients with UIP and OP subtypes of RA-ILD by two-dimensional gel electrophoresis (2-DE) and liquid spectrometry (LC−MS/MS). They found that in BALF, the immunoglobulin k chain level in UIP was significantly higher than that in OP. In addition, α-1 antitrypsin, CRP, haptoglobin β, and surfactant protein A were higher in OP than in UIP. This suggests that these proteins have different pathological mechanisms in different subtypes of RA-ILD. Interestingly, there was no significant difference in gelsolin in BALF, but C-gelsolin and N-gelsolin were significantly increased in UIP (12), which was also observed by Oikonomou et al. in animal models. It is speculated that C-gelsolin and N-gelsolin are gelsolin fragments mediated by caspase-3 (38). Proteomic studies can help to explore the specific molecular mechanism and solve related clinical problems by analyzing RA-ILD with different imaging manifestations.

Proteomics is also of great significance for diagnosing or monitoring RA-ILD. Wu et al. calculated the correlation between DEPs and clinical lung function indicators using a linear regression model and found that two proteins, paired immunoglobulin-like type two receptor-associated neural protein (PIANP) and secretory leukocyte peptidase inhibitor (SLPI), were related to the percentage of carbon monoxide diffusion capacity (13). This finding is significant for the clinical monitoring of disease progression in RA-ILD patients, but its specific practical effect needs to be verified in a large clinical cohort. In addition to this traditional method, Ma et al. used matrix-assisted laser desorption/ionization time-of-flight mass spectrometry (MALDI-TOF-MS) and found an overall change trend of serum protein changes between RA-non-ILD and RA-ILD patients, rather than quantifying the changes in each specific protein. Thirteen protein peaks were detected to be downregulated in RA-ILD patients, and the mass-to-charge value of protein peaks was used to establish the best tree model to distinguish RA-ILD from RA, with a sensitivity of 86.36%, a specificity of 84.09%, and an area under the ROC curve of 0.856 (14). However, the authors did not perform functional analysis of specific DEPs in protein peaks but verified that the model can be established using the overall change trend of proteins to distinguish whether RA patients are complicated with ILD.

In addition, proteomics is also an efficient technology for basic research to explore the treatment of RA-ILD. Wu et al. compared 98 DEPs between IPF and healthy controls. Sixteen proteins increased, while five decreased, and showed similar trends of change in RA-ILD and IPF compared with various control groups. However, four proteins were increased in RA-ILD but not in IPF (13). The results suggest that the pathogenesis of ILD in RA patients is similar to that in IPF patients, but there are also significant differences. These differences in proteins suggest that in the clinical treatment of RA-ILD, in addition to anti-pulmonary fibrosis treatment similar to IPF, other treatments for RA-specific lesions, such as specific autoantibodies and autoinflammation, should also be considered. In the same experiment, gene set enrichment analysis (GSEA) was performed on 234 DEPs between RA and RA-ILD. Signaling receptor binding, extracellular matrix, and negative regulation of proteolysis may play an important role in RA-ILD (13). These may be alternative drug targets for studying RA-ILD patients to strengthen anti-RA-based therapy.

### 2.2 Proteomics studies in SSc-ILD

SSc is characterized by immune dysregulation leading to inflammation and fibrosis of the skin and multiple internal organs (39). ILD is the most common and serious pulmonary complication of SSc, occurring in 47.0 to 66.4% of SSc patients and accounting for 35% of SSc-related mortality (40, 41). The pathogenesis of SSc-ILD may be multifactorial and is not fully understood.

Rottoli et al. compared the BALF protein composition in SSc-ILD, IPF, and sarcoidosis and found similar changes in many kinds of proteins among the three. Most plasma proteins (complement C3B, transthyretin, A-1-B glycoprotein, and serum retinol-binding protein (SRBP)) were the most highly expressed in sarcoidosis, followed by SSc-ILD, and IPF had the lowest expression. However, locally produced low molecular weight proteins, such as galectin 1, ubiquitin, and thioredoxin peroxidase 2, were more abundant in IPF (15). These findings suggest that the pathological mechanism of SSc-ILD differs from that of IPF. IPF is a generalized fibrotic disease confined to the lungs, while SSc-ILD is a local manifestation of systemic immune-inflammatory disease. In fibrotic lesions, the overexpression of extracellular matrix (ECM) proteins is considered a molecular marker of fibrosis (42). Mullenbrock et al. used LC−MS to specifically detect the protein composition of ECM in the lungs of patients with SSc-ILD and IPF. It was found that the soluble matrix proteins in the ECM of the lungs of SSc-ILD and IPF patients were significantly different compared to those in the healthy control group, but there were no differences in insoluble matrix proteins (25). Interestingly, SSc-ILD and IPF had a high overlap of lung ECM, especially several proteins related to fibrogenesis, such as PLOD2, LUM, POSTN, IGFBP5, and GREM1. The above results suggest that ECM protein signatures may be more reflective of fibrosis and less likely to indicate other SSc-associated pathologies (25).

In addition, Landi’s team found that 14-3-3ϵ was increased in the BALF proteome of SSc-ILD patients compared with nonsmokers (18, 20). In inflammatory and autoimmune diseases, 14-3-3-ϵ, as a component of the TNFR2 complex, restricts the activation of NF-κB through PI3K/Akt/mTOR signaling and stimulates the activation of C/EBP-β, thus guiding the plasticity of macrophages (43). However, studies on 14-3-3ϵ in pulmonary fibrosis, especially in CTD-ILD, are scarce. Similarly, the level of transthyretin in the BALF of patients with SSc-ILD was elevated, as detected by proteomic methods (15, 18, 20). One study found that transthyretin stimulates the production of collagen I and immunoglobulin-binding proteins in fibroblasts, which participate in endoplasmic reticulum stress activation and profibrosis through mitochondrial oxidative stress in cardiac amyloidosis (44). However, its function in CTD-ILD needs further confirmation. In addition to using BALF as a sample, Ryu et al. performed proteomic detection of extracellular vesicles (EVs) precipitated in plasma by LC-MS. The results showed that EVs from SSc-ILD patients contained significantly higher levels of MT-A TP6, and its protein was mainly involved in platelet activation, cell adhesion, and immune responses (23).

Larsen et al. conducted an interesting study on the detection modes. They compared the proteomics results of fibroblasts obtained from BALF and biopsies from SSc-ILD patients and found only three DEPs, indicating that BALF and biopsy fibroblast cultures from SSc-ILD patients were similar in protein composition (17). To some extent, we could use noninvasive BALF to replace biopsied active SSc-ILD-associated proteomic results.

Lung tissue is also an ideal sample type for CTD-ILD proteomic studies. Previous studies have demonstrated a genetic association between connective tissue growth factor (CTGF) and SSc (45, 46). This suggests that subclinical pathologic changes take place in the lungs of SSc patients even without established ILD. In addition, it was found that the response of the proteome of lung fibroblasts in non-ILD SSc patients had an excessive response to CTGF (24). This suggests that subclinical pathologic changes take place in the lungs of SSc patients even without established ILD. In addition to lung tissue or BALF for proteomic study samples from SSc-ILD patients, serum samples with a more extensive and convenient clinical application can be used. Van Bon et al. adopted the SELDI-TOF-MS examination of plasma from SSc patients with and without ILD and showed that S100A8 (calprotectin) levels were significantly increased in patients with SSc-ILD (21), which was consistent with the findings by Fietta et al. using BALF (19). Van Bon’s team also isolated plasmacyte-like dendritic cells from patients’ peripheral blood and used SELDITOF MS to analyze the whole proteome of the supernatant after cell lysis. It was found that SSc patients with higher CXCL4 levels in plasmacyte-like dendritic cell supernatant developed ILD significantly earlier, with a relative decline in forced vital capacity of over 30%, significantly faster decline in lung carbon monoxide diffusion capacity, and bilateral fibrosis on CT (22). Therefore, for long-term follow-up of SSc patients, detecting S100A8 and CXCL4 levels in plasmacytoid dendritic cell supernatant may be an efficient and convenient way to assess the risk of ILD.

The comorbidity rate of ILD in SSc patients is high, but there is still a lack of effective drug treatments, and the therapeutic mechanism of drugs is unclear. Notably, Shirahama et al., through 2-D gel electrophoresis of BALF, found that the number of protein species in SSc patients with ILD was greater than that in patients without ILD. In addition, five proteins were increased, while nine were decreased in patients with SSc-ILD. Specifically, glutathione S-transferase (GST) was increased in each patient with SSc-ILD compared with those without ILD (16). This result is consistent with the findings of Fietta et al. (19). Meanwhile, He et al. reported GST levels in bleomycin-induced pulmonary fibrosis mouse models (47). Strange et al. confirmed that GST contributes to the protection of biological macromolecules from oxidative stress by repairing damage to membrane phospholipids and inhibiting the induction of microsomal peroxidation (48). This point may provide evidence to support the treatment of pulmonary fibrosis with GST inhibitors. Sun et al. decellularized lung tissues from patients with SSc-ILD to obtain the lung cytoskeleton. The protein composition of the cytoskeleton was examined using the bottom-up label-free LC−MS quantitation technique, and periostin in SSc-ILD was found to be similar to previously reported changes in decellularized IPF lungs (49). However, the changes in Fibulin 3, Tinag-like 1, and Elastin were more significant in SSc-ILD (27), and drugs targeting these proteins may be considered SSC-ILD-specific therapies.

### 2.3 Proteomics studies in idiopathic inflammatory myopathies

Idiopathic inflammatory myopathy (IIM) is an autoimmune disease characterized by skeletal muscle weakness and inflammation, including DM, PM, anti-synthetase syndrome (ASS), and other subtypes. The skeletal muscle is usually affected, and lung changes are represented by ILD, which is the leading cause of death (50). In order to better explore the possible mechanism between the development of ILD in IIM patients, Fernandes-Cerqueira et al. performed proteomic analysis of peripheral serum from IIM and IIM-ILD patients by LC−MS/MS analysis. Among them, the abundance of Fc-agalactosylated glycan of IgG is increased in IIM patients compared to the general population. Intraindividual normalization of the main agalactosylated glycan (FA2) of IgG1 vs FA2-IgG2 was used to distinguish IIM-ILD from IIM-nonILD, which showed that the area under the curve (AUC) of this standard was 88 ± 6%. Moreover, the increase in Fc-agalactosylated glycan was not correlated with other extramuscular manifestations of IIM, further suggesting that the overexpression of Fc-agalactosylated glycan has a lung-specific propensity (28). In the same study, Fernandes-Cerqueira et al. performed a subgroup analysis of IIM with positive anti-Jo1 autoantibodies in the same group of samples and found that the abundance of Fc-glycan contained in JO1-specific IgG was lower than that of total IgG; that is, the proportion of Fc-agalactosylated glycan increased (28). This is consistent with the conclusion of previous studies that anti-Jo1 autoantibodies are common in IIM with ILD (51, 52).

In addition, Passadore et al. used 2-DE and LC−MS/MS to analyze the BALF proteomics of DM/PM patients with ILD. The samples were compared with those of ASS patients with anti-Jo1+ and ILD patients with myositis overlap syndrome. There were 24 specific protein spots among the three groups: nine spots were only present in DM/PM-ILD, three in ASS-ILD, and 12 in myositis overlap syndrome with ILD (29). In this experiment, the authors found that gelsolin was also increased in DM/PM-ILD, which was similar to that found by Suhara et al. in RA-ILD (12). However, Passadore et al. have a different understanding of the role of gelsolin in ILD and are more inclined to believe that gelsolin may maintain the inherent antimicrobial activity of the airway surface (53) and improve airway mucus viscosity by degrading a large amount of filamentous actin released by dead cells during inflammation (54). Similar to the findings of Fietta et al. using BALF of patients with SSc-ILD (19), calgranulin B was also increased in ILD patients with myositis overlap syndrome. Studies have shown that calgranulin B plays a role in endothelial inflammation and participates in the recruitment of white blood cells to the inflammatory site (55, 56), and its expression is related to the inflammatory activity of systemic vasculitis (57). These findings suggest that there may be a common pathological pathway among different CTD-ILD subtypes, but the role of various proteins needs further classification research.

### 2.4 Proteomics studies in other subtypes of CTD-ILD

Primary Sjögren’s syndrome is a chronic inflammatory autoimmune disease of unknown origin that particularly affects the tear and salivary glands (58). Li et al. also examined serum proteomics in patients with primary Sjogren’s syndrome (pSS) complicated with ILD. Seven protein peaks were significantly different between pSS-ILD and pSS. There were three peaks with ILD high expression and the rest with ILD low expression. Similar to the findings of Ma et al. (14), three differential protein peaks were selected to form a diagnostic model, with a sensitivity of 84% and a specificity of 85.7% (59). These results suggest that searching differential protein peaks using combinations of proteomics and statistical screening can effectively identify alternative diagnostic models of diseases.

Some proteomic studies have not been subdivided into subtypes of CTD-ILD, and the results are of great concern. For instance, cell adhesion molecule 1 (CADM1) was found to be downregulated in CTD-ILD (30). It has been suggested that it is a key adhesion receptor regulating human lung mast cells (HLMCs) and primary human lung fibroblasts (30, 60). SIL1 in DEPs is a nucleotide exchange factor for endoplasmic reticulum (ER) heat shock proteins in eukaryotic cells (61). ER stress is associated with various fibrotic diseases, including cystic fibrosis and idiopathic pulmonary fibrosis (62, 63). N-sulfoglucosamine sulfohydrolase (SGSH) has been reported to be involved in the desulfation of glycosaminoglycan chains on proteoglycans (64). Glycosaminoglycans are important components of lung ECM turnover, and abnormalities in the ECM are one of the main pathologies of pulmonary fibrosis (65). However, the pathological mechanism of the above DEPs in CTD-ILD has not been elucidated. This is also the most important difference between nonoffset proteomics and traditional experiments targeting specific proteins, which can more comprehensively identify the types of proteins that may be involved in the disease and provide more comprehensive and powerful clues for related basic research.

In addition, the correlation between some proteins and the occurrence and development of diseases has been studied more clearly, which can help to diagnose or monitor clinical diseases. Ye et al. detected 132 DEPs in the BALF of CTD-ILD patients using an LC−MS proteomic method. Surfactant protein D (SP-D), a humoral molecule of the congenital immune system, was found (30, 66), and Hant et al. pointed out in their pathological studies that it reflected the status of pulmonary fibrosis and could be used as an alternative indicator to evaluate lung involvement (67). Meanwhile, multiple studies have found that increased SP-D is associated with ILD in patients with SSc, RA, and DM/PM using serum samples (32, 67, 68). The presence of SP-D was also found in the subsynovium and microvascular endothelium of the pannus of the diseased joints in RA patients and was more common than in osteoarthritis patients (69). These findings suggest that the SP-D protein may be involved in the common pathogenesis and development of multiple CTD-ILD, primarily related to fibrosis, and may play a role in extrapulmonary organs. Recently, Bowman et al. explored possible proteins involved in the development of progressive ILD by performing proteomic analysis of peripheral blood from patients with ILD other than IPF, including CTD-ILD. They selected 12 DEPs as biomarkers to construct a risk assessment model for the development of progressive ILD (71). The detection results of CTD-ILD proteomics may not only be used as a guide for targeted research. All DEPs can be divided into a single object for study, and they can also be combined through statistical methods to conduct more direct clinical value transformation.

## 3 Conclusion

**Figure 1:**
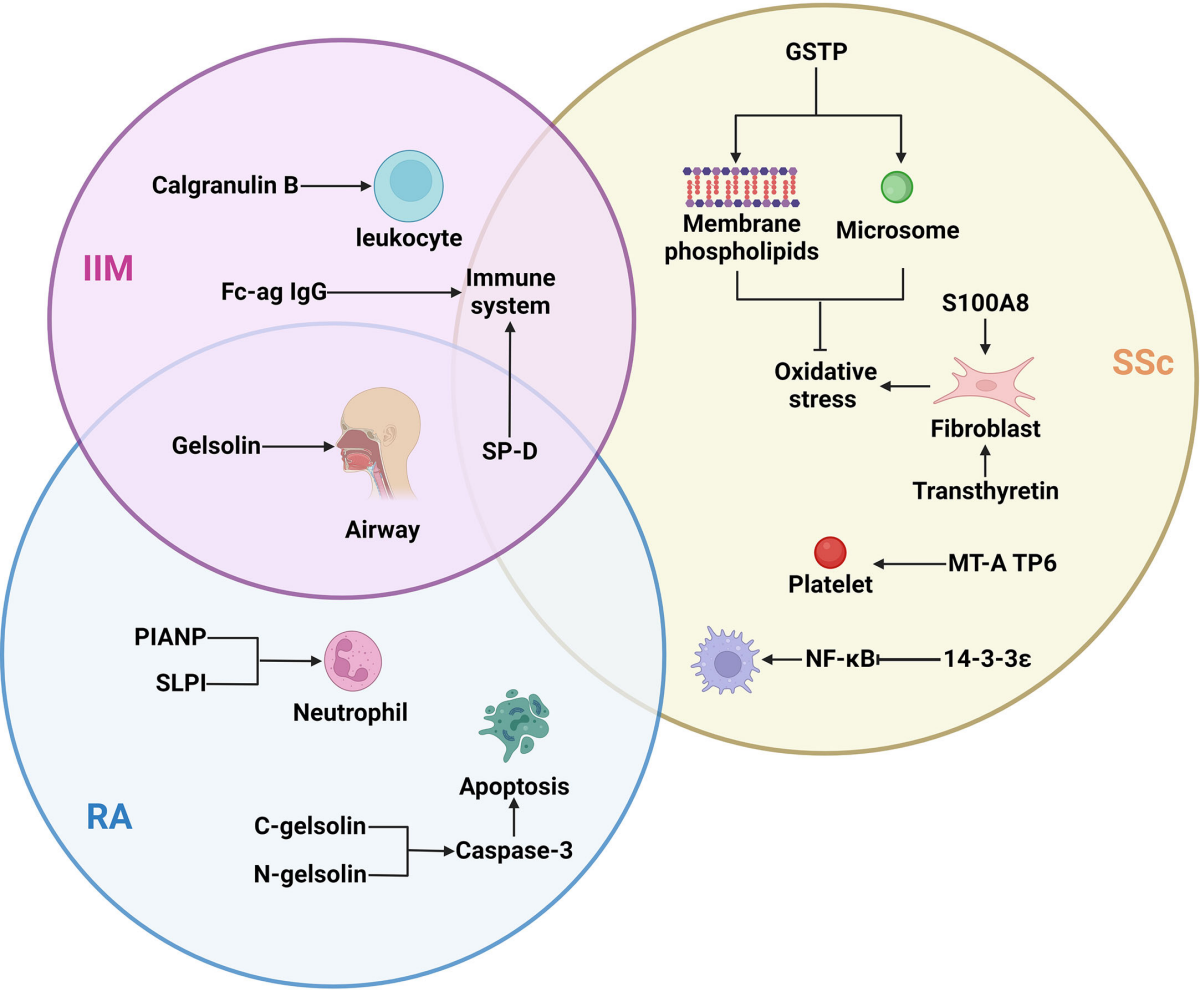
The important proteins of different subtypes of ILD and their target cells/organs: Different subtypes of ILD had different expression proteins, but SP-D differences were found in the three types of ILD, gelsolin differences were found in IIM and RA. IIM, idiopathic inflammatory myopathies; Fc-ag IgG, Fc-agalactosylated glycan of IgG; SP-D, surfactant protein D; SSc, systemic sclerosis; GSTP, glutathione S-transferase P; RA, rheumatoid arthritis; PIANP, paired immunoglobulin-like type two receptor-associated neural protein; SLPI, secretory leukocyte peptidase inhibitor.

Different CTD-ILD subtypes have different proteomic changes (**Figure 1**). The development of proteomic detection technology can help obtain relevant data from various samples to the maximum extent, explore specific pathogenesis, and search for clinical diagnosis and treatment biomarkers. However, uncertainties persist about the proteomic detection of many types of CTD-ILD, the changes in the protein composition of CTD-ILD patients before and after treatment, and the specific molecular mechanism of the participation of specific kinds of proteins in CTD-ILD. These questions need to be answered by future proteomic studies based on larger sample cohorts, prospective clinical studies, and sufficient clinical evidence.

## Author contributions

YLW and YHL wrote the review. CYT and YL contributed to the theme and structure of the review. YZ, JW, LC, XPL, and TW contributed to the literature search and summary. CYT, YL, and YBL made important modifications to important intellectual content. All authors read and approved the final manuscript.

## References

[1] Shum AK Alimohammadi M Tan CL Cheng MH Metzger TC Law CS. Bpifb1 is a lung-specific autoantigen associated with interstitial lung disease. Sci Trans Med (2013) 5(206):206ra139. doi: 10.1126/scitranslmed.3006998 PMC3882146 24107778

[2] Nakshbandi G Moor CC Wijsenbeek MS. Home monitoring for patients with ild and the covid-19 pandemic. Lancet Respir Med (2020) 8(12):1172–4. doi: 10.1016/s2213-2600(20)30452-5 PMC7567485 33075296

[3] Vij R Strek ME. Diagnosis and treatment of connective tissue disease-associated interstitial lung disease. Chest (2013) 143(3):814–24. doi: 10.1378/chest.12-0741 PMC3590889 23460159

[4] Barnes H Holland AE Westall GP Goh NS Glaspole IN. Cyclophosphamide for connective tissue disease-associated interstitial lung disease. Cochrane Database systematic Rev (2018) 1(1):Cd010908. doi: 10.1002/14651858.CD010908.pub2 PMC6491200 29297205

[5] Demoruelle MK Mittoo S Solomon JJ. Connective tissue disease-related interstitial lung disease. Best Pract Res Clin Rheumatol (2016) 30(1):39–52. doi: 10.1016/j.berh.2016.04.006 27421215

[6] Singh S Collins BF Sharma BB Joshi JM Talwar D Katiyar S. Interstitial lung disease in india. the results of a prospective registry. Am J Respir Crit Care Med (2017) 195(6):801–13. doi: 10.1164/rccm.201607-1484°C 27684041

[7] Steen VD Medsger TA. Changes in causes of death in systemic sclerosis, 1972-2002. Ann rheumatic Dis (2007) 66(7):940–4. doi: 10.1136/ard.2006.066068 PMC1955114 17329309

[8] Olson AL Swigris JJ Sprunger DB Fischer A Fernandez-Perez ER Solomon J. Rheumatoid arthritis-interstitial lung disease-associated mortality. Am J Respir Crit Care Med (2011) 183(3):372–8. doi: 10.1164/rccm.201004-0622°C PMC5450769 20851924

[9] Long K Danoff SK. Interstitial lung disease in polymyositis and dermatomyositis. Clinics chest Med (2019) 40(3):561–72. doi: 10.1016/j.ccm.2019.05.004 31376891

[10] Scherp P Ku G Coleman L Kheterpal I. Gel-based and gel-free proteomic technologies. Methods Mol Biol (Clifton NJ) (2011) 702:163–90. doi: 10.1007/978-1-61737-960-4_13 21082402

[11] Aslam B Basit M Nisar MA Khurshid M Rasool MH. Proteomics: Technologies and their applications. J chromatographic Sci (2017) 55(2):182–96. doi: 10.1093/chromsci/bmw167 28087761

[12] McInnes IB Schett G. The pathogenesis of rheumatoid arthritis. New Engl J Med (2011) 365(23):2205–19. doi: 10.1056/NEJMra1004965 22150039

[13] Spagnolo P Lee JS Sverzellati N Rossi G Cottin V. The lung in rheumatoid arthritis: Focus on interstitial lung disease. Arthritis Rheumatol (Hoboken NJ) (2018) 70(10):1544–54. doi: 10.1002/art.40574 29806092

[14] Doyle TJ Dellaripa PF Batra K Frits ML Iannaccone CK Hatabu H. Functional impact of a spectrum of interstitial lung abnormalities in rheumatoid arthritis. Chest (2014) 146(1):41–50. doi: 10.1378/chest.13-1394 24305643 PMC4077411

[15] Raimundo K Solomon JJ Olson AL Kong AM Cole AL Fischer A. Rheumatoid arthritis-interstitial lung disease in the united states: Prevalence, incidence, and healthcare costs and mortality. J Rheumatol (2019) 46(4):360–9. doi: 10.3899/jrheum.171315 30442831

[16] Wu X Jeong Y Poli de Frías S Easthausen I Hoffman K Oromendia C. Serum proteomic profiling of rheumatoid arthritis-interstitial lung disease with a comparison to idiopathic pulmonary fibrosis. Thorax (2022). doi: 10.1136/thorax-2021-217822 PMC9976796 35907639

[17] Ascherman DP. Interstitial lung disease in rheumatoid arthritis. Curr Rheumatol Rep (2010) 12(5):363–9. doi: 10.1007/s11926-010-0116-z 20628839

[18] Suhara K Miyazaki Y Okamoto T Ishizuka M Tsuchiya K Inase N. Fragmented gelsolins are increased in rheumatoid arthritis-associated interstitial lung disease with usual interstitial pneumonia pattern. Allergology Int (2016) 65(1):88–95. doi: 10.1016/j.alit.2015.08.002 26666486

[19] Oikonomou N Thanasopoulou A Tzouvelekis A Harokopos V Paparountas T Nikitopoulou I. Gelsolin expression is necessary for the development of modeled pulmonary inflammation and fibrosis. Thorax (2009) 64(6):467–75. doi: 10.1136/thx.2008.107946 19213772

[20] Ma D Liang N Zhang L. Establishing classification tree models in rheumatoid arthritis using combination of matrix-assisted laser Desorption/Ionization time-of-Flight mass spectrometry and magnetic beads. Front Med (2021) 8:609773. doi: 10.3389/fmed.2021.609773 PMC7943484 33718399

[21] Cottin V Brown KK. Interstitial lung disease associated with systemic sclerosis (Ssc-ild). Respir Res (2019) 20(1):13. doi: 10.1186/s12931-019-0980-7 30658650 PMC6339436

[22] Davidsen JR Miedema J Wuyts W Kilpeläinen M Papiris S Manali E. Economic burden and management of systemic sclerosis-associated interstitial lung disease in 8 European countries: The buildup Delphi consensus study. Adv Ther (2021) 38(1):521–40. doi: 10.1007/s12325-020-01541-5 PMC7854393 33156462

[23] Tyndall AJ Bannert B Vonk M Airò P Cozzi F Carreira PE. Causes and risk factors for death in systemic sclerosis: A study from the eular scleroderma trials and research (Eustar) database. Ann rheumatic Dis (2010) 69(10):1809–15. doi: 10.1136/ard.2009.114264 20551155

[24] Rottoli P Magi B Perari MG Liberatori S Nikiforakis N Bargagli E. Cytokine profile and proteome analysis in bronchoalveolar lavage of patients with sarcoidosis, pulmonary fibrosis associated with systemic sclerosis and idiopathic pulmonary fibrosis. Proteomics (2005) 5(5):1423–30. doi: 10.1002/pmic.200301007 15761959

[25] Xiao R Liu FY Luo JY Yang XJ Wen HQ Su YW. Effect of small interfering rna on the expression of connective tissue growth factor and type I and iii collagen in skin fibroblasts of patients with systemic sclerosis. Br J Dermatol (2006) 155(6):1145–53. doi: 10.1111/j.1365-2133.2006.07438.x 17107381

[26] Mullenbrock S Liu F Szak S Hronowski X Gao B Juhasz P. Systems analysis of transcriptomic and proteomic profiles identifies novel regulation of fibrotic programs by mirnas in pulmonary fibrosis fibroblasts. Genes (2018) 9(12). doi: 10.3390/genes9120588 PMC6316743 30501089

[27] Landi C Bargagli E Carleo A Refini RM Bennett D Bianchi L. Bronchoalveolar lavage proteomic analysis in pulmonary fibrosis associated with systemic sclerosis: S100a6 and 14-3-3ϵ as potential biomarkers. Rheumatol (Oxford England) (2019) 58(1):165–78. doi: 10.1093/rheumatology/key223 30239835

[28] Landi C Bargagli E Bianchi L Gagliardi A Carleo A Bennett D. Toward a functional proteomics approach to the comprehension of idiopathic pulmonary fibrosis, sarcoidosis, systemic sclerosis and pulmonary langerhans cell histiocytosis. J Proteomics (2013) 83:60–75. doi: 10.1016/j.jprot.2013.03.006 23528693

[29] Fu W Hu W Yi YS Hettinghouse A Sun G Bi Y. Tnfr2/14-3-3ϵ signaling complex instructs macrophage plasticity in inflammation and autoimmunity. J Clin Invest (2021) 131(16). doi: 10.1172/jci144016 PMC8363273 34185706

[30] Martínez-Martínez E Fernández-Irigoyen J Santamaría E Nieto ML Bravo-San Pedro JM Cachofeiro V. Mitochondrial oxidative stress induces cardiac fibrosis in obese rats through modulation of transthyretin. Int J Mol Sci (2022) 23(15). doi: 10.3390/ijms23158080 PMC9330867 35897655

[31] Ryu C Walia A Ortiz V Perry C Woo S Reeves BC. Bioactive plasma mitochondrial DNA is associated with disease progression in scleroderma-associated interstitial lung disease. Arthritis Rheumatol (Hoboken NJ) (2020) 72(11):1905–15. doi: 10.1002/art.41418 PMC8081728 32602227

[32] Larsen K Malmström J Wildt M Dahlqvist C Hansson L Marko-Varga G. Functional and phenotypical comparison of myofibroblasts derived from biopsies and bronchoalveolar lavage in mild asthma and scleroderma. Respir Res (2006) 7(1):11. doi: 10.1186/1465-9921-7-11 16430780 PMC1386661

[33] Fonseca C Lindahl GE Ponticos M Sestini P Renzoni EA Holmes AM. A polymorphism in the ctgf promoter region associated with systemic sclerosis. New Engl J Med (2007) 357(12):1210–20. doi: 10.1056/NEJMoa067655 17881752

[34] Zhao W Yue X Liu K Zheng J Huang R Zou J. The status of pulmonary fibrosis in systemic sclerosis is associated with Irf5, Stat4, Irak1, and ctgf polymorphisms. Rheumatol Int (2017) 37(8):1303–10. doi: 10.1007/s00296-017-3722-5 28434122

[35] Bogatkevich GS Ludwicka-Bradley A Singleton CB Bethard JR Silver RM. Proteomic analysis of ctgf-activated lung fibroblasts: Identification of Iqgap1 as a key player in lung fibroblast migration. Am J Physiol Lung Cell Mol Physiol (2008) 295(4):L603–11. doi: 10.1152/ajplung.00530.2007 PMC2575950 18676875

[36] van Bon L Cossu M Loof A Gohar F Wittkowski H Vonk M. Proteomic analysis of plasma identifies the toll-like receptor agonists S100a8/A9 as a novel possible marker for systemic sclerosis phenotype. Ann rheumatic Dis (2014) 73(8):1585–9. doi: 10.1136/annrheumdis-2013-205013 24718960

[37] Fietta A Bardoni A Salvini R Passadore I Morosini M Cavagna L. Analysis of bronchoalveolar lavage fluid proteome from systemic sclerosis patients with or without functional, clinical and radiological signs of lung fibrosis. Arthritis Res Ther (2006) 8(6):R160. doi: 10.1186/ar2067 17044913 PMC1794502

[38] van Bon L Affandi AJ Broen J Christmann RB Marijnissen RJ Stawski L. Proteome-wide analysis and Cxcl4 as a biomarker in systemic sclerosis. New Engl J Med (2014) 370(5):433–43. doi: 10.1056/NEJMoa1114576 PMC4040466 24350901

[39] Shirahama R Miyazaki Y Okamoto T Inase N Yoshizawa Y. Proteome analysis of bronchoalveolar lavage fluid in lung fibrosis associated with systemic sclerosis. Allergology Int Off J Japanese Soc Allergology (2010) 59(4):409–15. doi: 10.2332/allergolint.10-OA-0176 20962569

[40] He N Bai S Huang Y Xing Y Chen L Yu F. Evaluation of glutathione s-transferase inhibition effects on idiopathic pulmonary fibrosis therapy with a near-infrared fluorescent probe in cell and mice models. Analytical Chem (2019) 91(8):5424–32. doi: 10.1021/acs.analchem.9b00713 30869868

[41] Strange RC Jones PW Fryer AA. Glutathione s-transferase: Genetics and role in toxicology. Toxicol Lett (2000) 112-113:357–63. doi: 10.1016/s0378-4274(99)00230-1 10720752

[42] Booth AJ Hadley R Cornett AM Dreffs AA Matthes SA Tsui JL. Acellular normal and fibrotic human lung matrices as a culture system for in vitro investigation. Am J Respir Crit Care Med (2012) 186(9):866–76. doi: 10.1164/rccm.201204-0754°C PMC3530219 22936357

[43] Sun H Zhu Y Pan H Chen X Balestrini JL Lam TT. Netrin-1 regulates fibrocyte accumulation in the decellularized fibrotic sclerodermatous lung microenvironment and in bleomycin-induced pulmonary fibrosis. Arthritis Rheumatol (Hoboken NJ) (2016) 68(5):1251–61. doi: 10.1002/art.39575 PMC5547894 26749424

[44] Jee AS Parker MJS Bleasel JF Troy LK Lau EM Jo HE. Diagnosis of myositis-associated interstitial lung disease: Utility of the myositis autoantibody line immunoassay. Respir Med (2021) 187:106581. doi: 10.1016/j.rmed.2021.106581 34454312

[45] Fernandes-Cerqueira C Renard N Notarnicola A Wigren E Gräslund S Zubarev RA. Patients with anti-Jo1 antibodies display a characteristic igg fc-glycan profile which is further enhanced in anti-Jo1 autoantibodies. Sci Rep (2018) 8(1):17958. doi: 10.1038/s41598-018-36395-z 30560888 PMC6298993

[46] Ingegnoli F Lubatti C Ingegnoli A Boracchi P Zeni S Meroni PL. Interstitial lung disease outcomes by high-resolution computed tomography (Hrct) in anti-Jo1 antibody-positive polymyositis patients: A single centre study and review of the literature. Autoimmun Rev (2012) 11(5):335–40. doi: 10.1016/j.autrev.2011.09.007 21985773

[47] Liu Y Luo H Wang L Li C Liu L Huang L. Increased serum matrix metalloproteinase-9 levels are associated with anti-Jo1 but not anti-Mda5 in myositis patients. Aging Dis (2019) 10(4):746–55. doi: 10.14336/ad.2018.1120 PMC6675534 31440381

[48] Passadore I Iadarola P Di Poto C Giuliano S Montecucco C Cavagna L. 2-de and lc-Ms/Ms for a comparative proteomic analysis of balf from subjects with different subsets of inflammatory myopathies. J Proteome Res (2009) 8(5):2331–40. doi: 10.1021/pr800943t 19301896

[49] Weiner DJ Bucki R Janmey PA. The antimicrobial activity of the cathelicidin Ll37 is inhibited by f-actin bundles and restored by gelsolin. Am J Respir Cell Mol Biol (2003) 28(6):738–45. doi: 10.1165/rcmb.2002-0191°C 12600826

[50] Candiano G Bruschi M Pedemonte N Caci E Liberatori S Bini L. Gelsolin secretion in interleukin-4-Treated bronchial epithelia and in asthmatic airways. Am J Respir Crit Care Med (2005) 172(9):1090–6. doi: 10.1164/rccm.200409-1185°C 16100010

[51] Li C Li S Jia C Yang L Song Z Wang Y. Low concentration of S100a8/9 promotes angiogenesis-related activity of vascular endothelial cells: Bridges among inflammation, angiogenesis, and tumorigenesis? Mediators Inflammation (2012) 2012:248574. doi: 10.1155/2012/248574 PMC3363068 22685372

[52] Vogl T Ludwig S Goebeler M Strey A Thorey IS Reichelt R. Mrp8 and Mrp14 control microtubule reorganization during transendothelial migration of phagocytes. Blood (2004) 104(13):4260–8. doi: 10.1182/blood-2004-02-0446 15331440

[53] Viemann D Strey A Janning A Jurk K Klimmek K Vogl T. Myeloid-related proteins 8 and 14 induce a specific inflammatory response in human microvascular endothelial cells. Blood (2005) 105(7):2955–62. doi: 10.1182/blood-2004-07-2520 15598812

[54] Stefanski AL Tomiak C Pleyer U Dietrich T Burmester GR Dörner T. The diagnosis and treatment of sjögren's syndrome. Deutsches Arzteblatt Int (2017) 114(20):354–61. doi: 10.3238/arztebl.2017.0354 PMC5471601 28610655

[55] Li YH Sun XL He J Jia RL Yang DY Zhang XW. [Screening for serum specific biomarkers in patients with primary sjögren's syndrome and interstitial lung disease using proteomic fingerprint techniques]. Beijing da xue xue bao Yi xue ban = J Peking Univ Health Sci (2012) 44(2):240–3. 22516996

[56] Ye J Liu P Li R Liu H Pei W Ma C. Biomarkers of connective tissue disease-associated interstitial lung disease in bronchoalveolar lavage fluid: A label-free mass spectrometry-based relative quantification study. J Clin Lab Anal (2022) 36(5):e24367. doi: 10.1002/jcla.24367 35334492 PMC9102639

[57] Moiseeva EP Roach KM Leyland ML Bradding P. Cadm1 is a key receptor mediating human mast cell adhesion to human lung fibroblasts and airway smooth muscle cells. PloS One (2013) 8(4):e61579. doi: 10.1371/journal.pone.0061579 23620770 PMC3631237

[58] Ichhaporia VP Kim J Kavdia K Vogel P Horner L Frase S. Sil1, the endoplasmic-Reticulum-Localized bip Co-chaperone, plays a crucial role in maintaining skeletal muscle proteostasis and physiology. Dis Models Mech (2018) 11(5). doi: 10.1242/dmm.033043 PMC5992605 29666155

[59] Ruan J Liang D Yan W Zhong Y Talley DC Rai G. A small-molecule inhibitor and degrader of the Rnf5 ubiquitin ligase. Mol Biol Cell (2022) 33(13):ar120. doi: 10.1091/mbc.E22-06-0233 36074076 PMC9634977

[60] Dobrinskikh E Hennessy CE Kurche JS Kim E Estrella AM Cardwell J. Epithelial er stress enhances the risk of Muc5b associated lung fibrosis. Am J Respir Cell Mol Biol (2022) 68(1):62–74. doi: 10.1165/rcmb.2022-0252°C PMC9817917 36108173

[61] Harmatz P Muenzer J Ezgü F Dalén P Huledal G Lindqvist D. Chemically modified recombinant human sulfamidase (Sobi003) in mucopolysaccharidosis iiia patients: Results from an open, non-controlled, multicenter study. Mol Genet Metab (2022) 136(4):249–59. doi: 10.1016/j.ymgme.2022.06.008 35835061

[62] Caird R Williamson M Yusuf A Gogoi D Casey M McElvaney NG. Targeting of glycosaminoglycans in genetic and inflammatory airway disease. Int J Mol Sci (2022) 23(12). doi: 10.3390/ijms23126400 PMC9224208 35742845

[63] Lin Z Thorenoor N Wu R DiAngelo SL Ye M Thomas NJ. Genetic association of pulmonary surfactant protein genes, Sftpa1, Sftpa2, sftpb, sftpc, and sftpd with cystic fibrosis. Front Immunol (2018) 9:2256. doi: 10.3389/fimmu.2018.02256 30333828 PMC6175982

[64] Hant FN Ludwicka-Bradley A Wang HJ Li N Elashoff R Tashkin DP. Surfactant protein d and kl-6 as serum biomarkers of interstitial lung disease in patients with scleroderma. J Rheumatol (2009) 36(4):773–80. doi: 10.3899/jrheum.080633 19286849

[65] Avouac J Cauvet A Steelandt A Shirai Y Elhai M Kuwana M. Improving risk-stratification of rheumatoid arthritis patients for interstitial lung disease. PloS One (2020) 15(5):e0232978. doi: 10.1371/journal.pone.0232978 32384128 PMC7209254

[66] Lyu W Zhou Y Zhuang Y Liu Y Cao M Xin X. Surfactant protein d is associated with 3-month mortality of anti-Mda5 antibody-interstitial lung disease. Clin Exp Rheumatol (2020) 38(6):1068–74. doi: 10.1164/ajrccm-conference.2020.201.1_MeetingAbstracts.A1093 31994487

[67] Christensen AF Sorensen GL Junker K Revald PH Varnum C Sorensen FB. Localization of surfactant protein-d in the rheumatoid synovial membrane. APMIS Acta pathologica microbiologica immunologica Scandinavica (2018) 126(1):9–13. doi: 10.1111/apm.12785 29155458

[68] Bowman WS Newton CA Linderholm AL Neely ML Pugashetti JV Kaul B. Proteomic biomarkers of progressive fibrosing interstitial lung disease: A multicenter cohort analysis. Lancet Respir Med (2022) 10(6):593–602. doi: 10.1016/s2213-2600(21)00503-8 35063079 PMC9177713

[69] Lear TB Lockwood KC Larsen M Tuncer F Kennerdell JR Morse C. Kelch-like protein 42 is a profibrotic ubiquitin E3 ligase involved in systemic sclerosis. J Biol Chem (2020) 295(13):4171–80. doi: 10.1074/jbc.AC119.012066 PMC7105301 32071084

