# Supplementary material for: Proteomics: Potential techniques for discovering the pathogenesis of connective tissue diseases-interstitial lung disease
Source: Front Immunol. 2023 Mar 29;14:1146904. doi: 10.3389/fimmu.2023.1146904 (PMC10090492; doi:10.3389/fimmu.2023.1146904)
Supplement: Supplementary file 1 [file DataSheet_1.docx]

**Search strategy**

Regarding our criteria for searching and screening articles cited in articles, we mainly searched and screened articles in PubMed, Embase, OVID, and Web of Science based on subject headings: “connective tissue disease”, “interstitial lung disease”, “proteomics”, “pulmonary fibrosis”, “rheumatoid arthritis”, “systemic sclerosis", "scleroderma", "dermatomyositis", "sjogren syndrome", and "systemic lupus erythematosus" were searched for relevant studies published up to November 20, 2022. By reading the content of the article, we screened the studies that were in line with the theme of this review for induction and discussion.
